# Effect of Psychotropic Drugs on Development of Diabetes Mellitus in Patients With Alzheimer's Disease

**DOI:** 10.1097/MD.0000000000000919

**Published:** 2015-06-12

**Authors:** Ki Jung Chang, Chang Hyung Hong, Yunhwan Lee, Kang Soo Lee, Hyun Woong Roh, Joung Hwan Back, Young Ki Jung, Ki Young Lim, Jai Sung Noh, Hyun Chung Kim, Seong Hye Choi, Seong Yoon Kim, Duk L. Na, Sang Won Seo, Soojin Lee, Sang Joon Son

**Affiliations:** From the Department of Psychiatry, Ajou University School of Medicine (KJC, CHH, HWR, YKJ, ZKYL, JSN, SJS); Institute on Aging, Ajou University Medical Center (CHH, YL); Department of Preventive Medicine and Public Health, Ajou University School of Medicine, Suwon (YL); Department of Psychiatry, CHA University School of Medicine, CHA Hospital, Bundang (KSL); Health Insurance Policy Research Institute (JHB); Department of Psychiatry, National Medical Center, Seoul (HCK); Department of Neurology, Inha University College of Medicine, Incheon (SHC); Memory Impairment Center, Ajou University Hospital, Suwon (CHH); Department of Psychiatry, Asan Medical Center (SYK); Department of Neurology, Samsung Medical Center, Sungkyunkwan University School of Medicine (DLN, SWS); and Department of Medicare Administration, Backseok Arts University, Seoul, South Korea (SL).

## Abstract

We aimed to examine risk of diabetes mellitus (DM) among older adults with Alzheimer's disease receiving 3 types of psychotropic drugs, that is, antipsychotics, antidepressants, and sedative anxiolytics.

We retrospectively analyzed data from a hospital-based Clinical Research Center for Dementia of South Korea (CREDOS) study conducted between January 1, 2008 and December 31, 2012. Participants (n = 3042) with Alzheimer's disease were aged 65 or older and had no preexisting history of DM. Development of DM was identified using claims for initiating at least 1 prescription of antidiabetic medications or a diagnosis of DM during the follow-up period. Cox proportional hazards regression was used to demonstrate the Hazard ratio of DM in use of each psychotropic drug.

Among the 3042 participants, 426 patients (14.0%) developed DM, representing an incidence rate of 5.2/100 person-years during an average 2.9 years of follow-up period. Among the 3 types of psychotropic drugs, antipsychotic users had a significantly higher risk of DM (hazard ratio = 1.74, 95% confidence interval = 1.10, 2.76) than nonusers, after adjusting covariates. Antidepressants and sedative anxiolytics did not achieve statistical significance.

These results suggested that the diabetes risk was elevated in Alzheimer patients on antipsychotic treatment. Therefore, patients with Alzheimer's disease receiving antipsychotic treatment should be carefully monitored for the development of DM.

## INTRODUCTION

Patients with Alzheimer's disease are prescribed not only cholinesterase inhibitors, but also psychotropic drugs including antipsychotics, antidepressants, and sedative anxiolytics to relieve a variety of symptoms in clinical practice.^1,2^ For example, although any antipsychotics were not FDA-approved for the treatment of neuropsychiatric symptoms in dementia, the off-label use of antipsychotics to treat severe neuropsychiatric symptoms such as psychotic symptoms, agitation, and aggression in patients with dementia is increasing.^3,4^ It is often recommended to consider nonpharmacological before pharmacologic interventions.^5^ Nevertheless, limited efficacy of nonpharmacological interventions frequently leads to these pharmacological management.^5,6^ With the increasing use of psychotropic drugs in patients with Alzheimer's disease, an issue has recently been raised on the safety of these drugs.

The development of diabetes mellitus (DM) was one of the serious and chronic adverse effects among psychotropic drug-induced adverse events in the general population.^7^ Antipsychotics are of particular concern among 3 types of psychotropic drugs, since they cause DM, as well as serious cardiovascular disease, stroke, and increased mortality.^8^ Many studies have established that use of antipsychotics increases the risk of new onset DM in patients with schizophrenia and bipolar disorder.^9–12^ The U.S Food and Drug Administration (FDA) accordingly recommended baseline screening and routine ongoing monitoring of risk factors related to DM throughout antipsychotic therapy in antipsychotics users of all ages with a risk of DM.^3^ Researchers also reported that antidepressants might increase the prevalent risk of DM by significant weight gain and increased insulin resistance.^13,14^ Furthermore, although a few studies have been conducted compared to antipsychotics and antidepressants, a research has suggested an increased metabolic risk with the use of sedative anxiolytics.^15^

Interestingly above results in general population did not extend to patients with dementia. Contrary to expectations, some studies in demented patients did not demonstrate a direct effect of psychotropic drugs including antipsychotics, antidepressants and sedative anxiolytics on the development of DM.^16,17^ The reason for uncertainty in patients with dementia might be as follows. First, elderly patients with dementia tended toward weight loss than general population due to poor nutrition and increased energy expenditure.^18^ Considering this metabolic change in elderly patients with dementia might be important since it might affect the development of drug-associated DM.^19,20^ Second, there were methodological differences between previous studies, participants in previous studies were mixed with healthy older adults and patients with cognitive impairment including dementia. In addition, several studies did not control other comorbid medical conditions related to the development of DM.

Based on the above considerations, we investigated the effects of exposure to psychotropic drugs including antipsychotics, antidepressants, and sedative anxiolytics on the development of DM in patients with Alzheimer's disease adjusting BMI, comorbid medical conditions, and other covariates related DM.

## METHODS

### Data Source

We used 2 data sets to test our hypotheses: the multicenter study of dementia by Clinical Research Center for Dementia of South Korea (CREDOS) data, and the Health Insurance Review and Assessment Service (HIRA) data. First, the CREDOS data is a nationwide multicenter study designed to assess the occurrence and risk factors of cognitive disorders. The CREDOS study, registered on ClinicalTrials.gov (identifier; NCT 01198093), recruited patients with dementia from 31 university-affiliated hospitals in South Korea. A more detailed description of CREDOS is available elsewhere.^21,22^ Among the subjects of CREDOS study, we selected 5,080 older adults aged 65 or over who were diagnosed with Alzheimer's disease. We used health insurance claim data for which a review and an assessment were completed by the HIRA from January 1, 2008 to December 31, 2012 to confirm the development of DM and prescription of psychotropic drugs including antipsychotics, antidepressants, and sedative anxiolytics. The National Health Insurance (NHI) Claims Database of the Health Insurance Review and Assessment Service (HIRA) includes medical claims filed by medical institutions through the NHI that are compiled and referenced through the Electronic Data Interchange (EDI).

### Study Population

We retrospectively analyzed data for patients with the following inclusion criteria: aged 65 or over at the time of cohort entry; confirmed diagnosis of Alzheimer's disease in the CREDOS study at cohort entry by psychiatrists or neurologists. The diagnostic criteria for Alzheimer's disease were based on the National Institute of Neurological and Communicative Disorders and Territorial infarction-Alzheimer's Disease and Related Disorders Association (NINCDS-ADRDA)^23^ and a clinical dementia rating (CDR) score of 0.5–3. The study's exclusion criteria were as follows: first, history of hearing or visual impairment rendering participation in the interview difficult; second, neurologic disorder (eg, territorial infarction, intracranial hemorrhage, brain tumor, and hydrocephalus); third, psychiatric disorders (eg, schizophrenia, mental retardation, or mania); fourth, subjects with a mismatch between information in CREDOS study and claim data from EDI; and fifth, a preexisting history of DM, as evidenced by a medical claim for antidiabetics ordiagnosed DM by an *International Classification of Disease, Tenth Revision* (ICD-10) from E10 to E14 code before cohort entry. A total of 3,042 people participated in our study. Each subject in this study was tracked for the development of DM during exposure to psychotropic drugs from cohort entry to December 31, 2012. Development of DM was identified from claims for initiating at least 1 prescription of antidiabetic medication or diagnosis of DM at least once by ICD-10 from E10 to E14 code after cohort entry. This study was approved by the Institutional Review Boards of the participating centers, and informed consent was obtained from all subjects.

### Measurements

The recorded information included patient demographic characteristics (age, sex, and level of education) at cohort entry. The level of education was divided into elementary school or lower, middle-high school, college or higher. Medical comorbidities were predicted using the Charlson comorbidity index (CCI).^24^ CCI is based on disease severity; each condition is assigned a score of 1 to 6 depending on the risk of dying associated with that condition. Weights were assigned to 17 predetermined clinical conditions (adapted from the ICD-10 codes of Sundararajan's version) and summed. The extent of obesity was identified using body mass index (BMI). We categorized subjects into 5 groups consisting of underweight (BMI < 18.5 kg/m^2^), normal range (18.5 ≤ BMI ≤ 22.9 kg/m^2^), overweight-at risk (23.0 ≤ BMI ≤ 24.9 kg/m^2^), overweight-moderately obese (25.0 ≤ BMI ≤ 29.9 kg/m^2^), and overweight-severely obese (BMI ≥ 30 kg/m^2^). The severity of Alzheimer's disease was measured by the clinical dementia rating (CDR).^25^ A Korean version of the mini-mental state examination (MMSE) was used to evaluate cognitive function.^26^ A Korean study defined the cut-off point of the MMSE score while screening dementia as 17/18 points; the sensitivity and specificity of the findings were 91% and 86%, respectively.^27^ We assessed depression using the Korean version of the Geriatric Depression Scale-Short Form (GDS).^28^ A cut-off point of 8 has a sensitivity of 85% and a specificity of 69% for diagnosing a major depressive episode compared with the *Diagnostic and Statistical Manual of Mental Disorders*, Third Edition-Revised (DSM-III-R).^28^ We identified all prescriptions of antipsychotics, antidepressants, and sedative anxiolytics in each subject by HIRA data from January 1, 2008, which was the beginning day of CREDOS study, to cohort entry. We categorized subjects into 2 groups based on exposure to a psychotropic drug, that is, “user” and “non-user,” applying a dichotomous definition of drug exposure: at least 1 taking a psychotropic drug versus no taking a psychotropic drug.^29^

### Statistical Analysis

Categorical variables were reported as frequencies and percentages, while continuous variables were reported as the mean with standard deviation. Discrete variables were compared using *χ*^2^ tests. Independent Student *t* tests were used to compare characteristics between patients who did not develop DM and who eventually developed DM. The incidence rate of diabetes was calculated for the subjects at the end of follow-up. The incident rates for DM were estimated using the Kaplan–Meier method. Cox proportional hazards regression was created to estimate the Hazard ratio and 95% confidence intervals of the development of DM. All statistical analyses were performed by Statistical Analysis System (SAS) version 9.3 (SAS Institute Inc, Cary, NC).

## RESULTS

A total of 3,042 individuals were followed for an average of 2.9 years per each subject in this study. The general characteristics of the participants are shown in Table 1. The mean age of the participants was 76.7 ± 6.0 years. Out of the 3,042 participants, 888 (29.2%) were male and 2,154 (70.8%) were female; the male-to-female ratio was approximately 1:2.5. At cohort entry, 31.7% of patients received sedative anxiolytics, followed by antidepressants (7.5%) and antipsychotics (5.7%). Mean CCI score was 3.5 ± 1.8 and 23.2% of subjects were moderately to severely obese. A total of 83.4% subjects had a CDR of 0.5–1. Based on the distribution of CDR, subjects in this study mostly consisted of patients with mild Alzheimer's disease. Table 1 also presents comparisons of patient's characteristics according to whether DM eventually developed. Patients who developed DM had more comorbid conditions (*P* < 0.001) and were significantly more obese (*P* = 0.01). There were no significant differences in users of antipsychotics, antidepressants, and sedative anxiolytics at cohort entry who developed DM and did not develop DM.

**TABLE 1:**
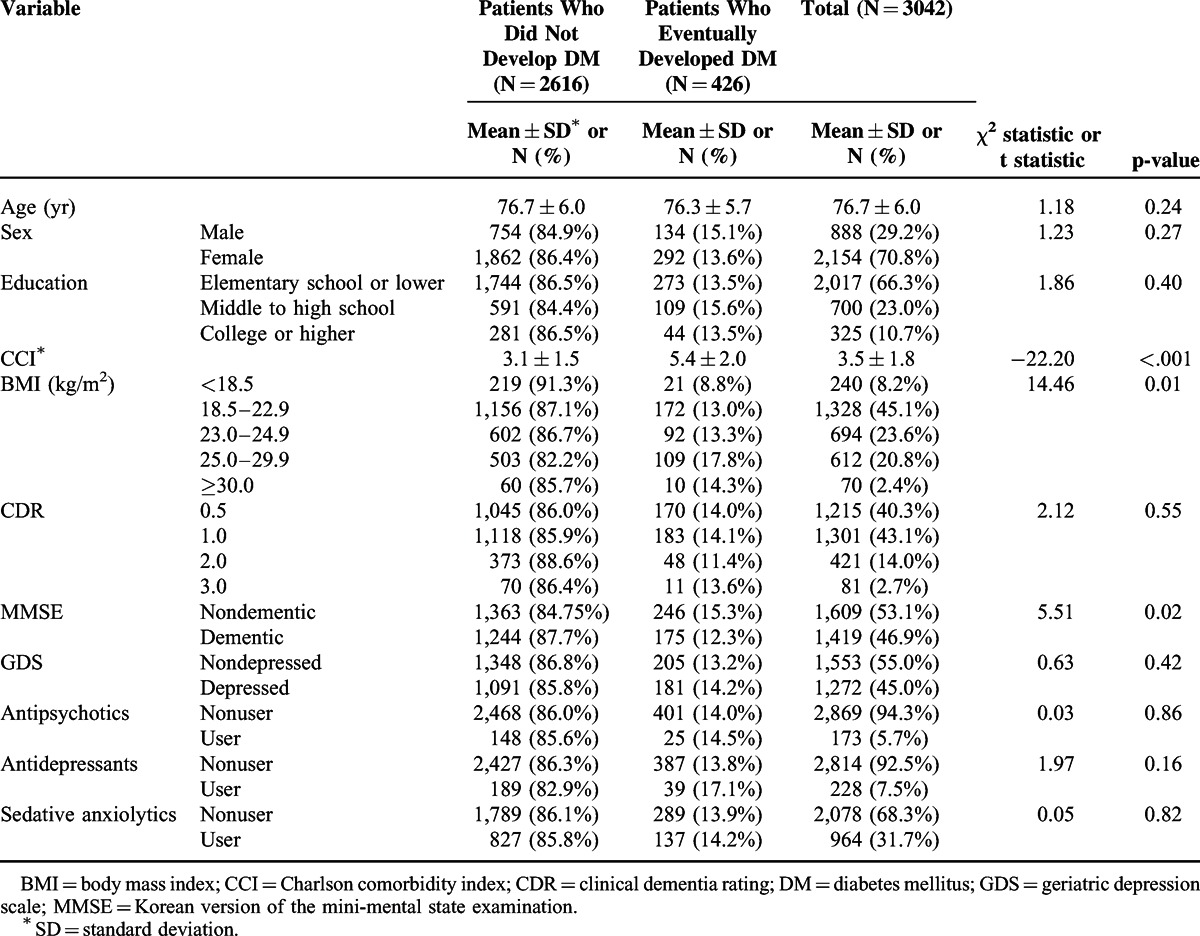
Baseline Characteristics of Study Participants by Diabetes Mellitus

**FIGURE 1:**
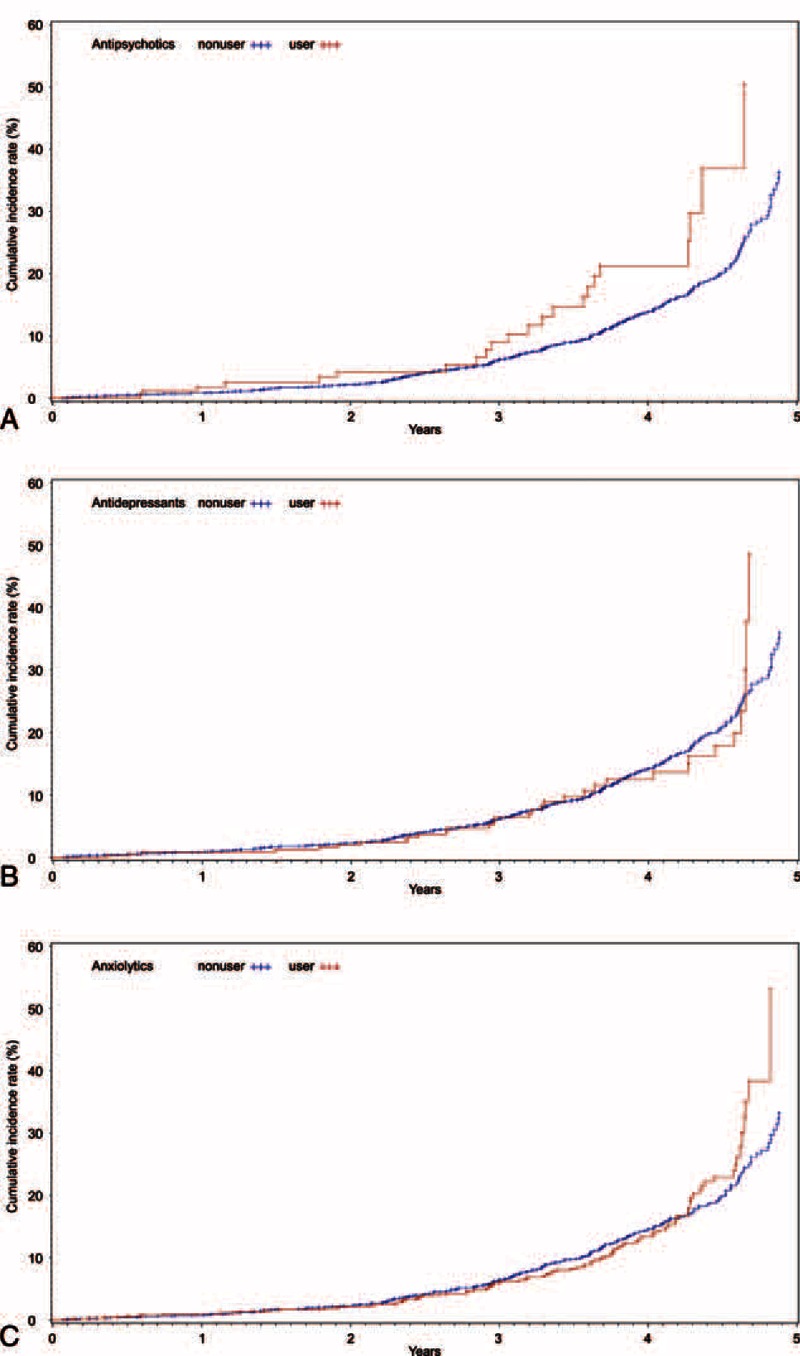
Fitted survival functions from the cox proportional hazards model predicting development of diabetes mellitus among patients with Alzheimer's disease while receiving each psychotropic drug.

Overall, 426 patients (14.0%) of the 3,042 participants were diagnosed with DM, representing an incidence rate of 5.2/100 person-years during the study follow-up period. Table 2 shows the results from the cox proportional hazards model predicting risk factors of DM during exposure to psychotropic drugs. Among the 3 types of psychotropic drugs, only antipsychotics were associated with the development of DM (hazard ratio = 1.74, 95% CI = 1.10, 2.76) after adjusting demographic factors (sex, age, education), CCI, BMI, CDR, MMSE, GDS. Diabetic risk in the patients who received antipsychotic prescription was 1.74 times higher than the nonuser group of antipsychotics in patients with Alzheimer's disease. Antidepressants and sedatives did not achieve statistical significance in patients with Alzheimer's disease. Figure 1 presents the survival functions from the cox proportional hazards model while receiving each psychotropic drug.

**TABLE 2:**
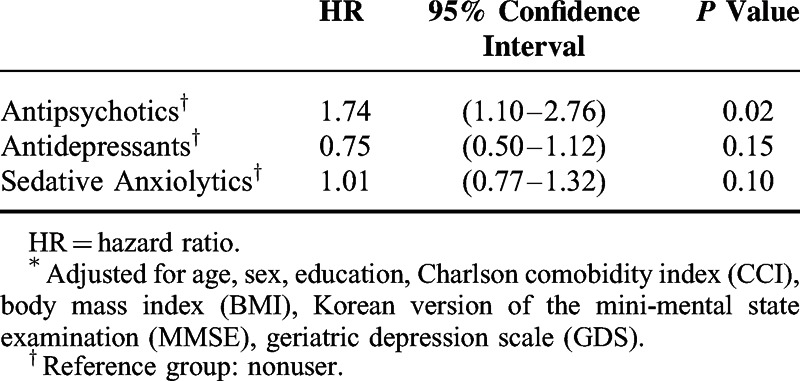
Adjusted Hazard Ratio With 95% Confidence Interval for Diabetes Mellitus Comparing Psychotropic Drugs From a Multivariate Cox Model^∗^

## DISCUSSION

Our data indicated that use of antipsychotics at baseline increased the risk of DM in patients with Alzheimer's disease during the study period. Patients with exposure to antipsychotics in particular had a 74% increased risk of DM during the follow-up period. Furthermore, other psychotropic drugs including antidepressants and sedative anxiolytics did not influence the development of DM inpatients with Alzheimer's disease.

Among the 3 types of psychotropic drugs prescribed in patients with Alzheimer's disease, we confirmed the association of antipsychotics on DM. Some studies of the relationship between development of DM and antipsychotic use in patients with dementia had contrasting results to our result on the risk of DM in geriatric patients with dementia.^16,17,30^ Micca et al conducted an analysis of patients with taking olanzapine, which were evaluated among 1,678 patients with dementia. Overall 2.1% patients with dementia developed DM and there were no significant differences between the olanzapine and placebo groups.^17^ However, authors presented limitations to their study, which was conducted on only 1 type of antipsychotics and omitted BMI data by approximately over 70% of participants. Additionally, since the average follow-up periods of the7 studies were from 2 to 6 months, it was a relatively short period to detect the development of DM during antipsychotics therapy. Another cross sectional study of 99 patients with Alzheimer's disease showed no differences in blood glucose concentration between patients with and without antipsychotics.^16^ However, the authors recommended caution in the interpretation of their results due to small sample size and since hyperglycemia does not exactly indicate the development of DM.

On the other hand, there were several reports that a risk of DM elevated in geriatric patients, which included patients with dementia, receiving antipsychotics.^31–33^ Erickson et al matched 13,075 participants aged 65 and older receiving antipsychotics with 65,375 patients receiving none. When CCI were added as a covariate in analysis of this study, the adjusted odds ratio was 1.32 for geriatric patients with versus without antipsychotics exposure.^33^ The CATIE-AD study was the prospective study to examine the effects and side effects of antipsychotics in 421 patients with only Alzheimer's disease. Although the power of this study was limited by the high discontinuation rate, it showed that antipsychotics lead to increase the metabolic complication such as weight gain-related DM.^32^ Previous results among patients with Alzheimer's disease have been inconclusive due to various methodological limitations and small sample size. Our results, however, corroborated the literature on the negative impact of antipsychotics on the development of DM.

The mechanism for the increasing risk of DM in patients with Alzheimer's disease taking antipsychotics remains unclear. Many studies suggested that the elevated risk of DM is secondary to weight gain and insulin resistance on receiving antipsychotics.^34–36^ Some researchers explained the lower than general population incidence rate of DM during antipsychotics, in patients with dementia, to the fact that those with dementia tended toward weight loss than weight gain.^19,20^ However, we found that the association between the use of antipsychotics and development of DM maintained after adjusting BMI. It means that we should focus on direct effect of antipsychotics on DM, not mediating effect of antipsychotics on weight change related to DM. For example, antipsychotics might exacerbate underlying pre-diabetes.^37^ Since baseline blood glucose levels were not available in this study, we unfortunately could not evaluate the presence of pre-diabetes at cohort entry.

We also found that other psychotropic drugs with the exception of antipsychotics were not associated with an increased risk of DM in patients with Alzheimer's disease. Some researchers suggested hypothesis that TCA and some kinds of SSRI causing weight gain might increase the risk of DM similar to antipsychotics.^7,13^ But, several researches produced inconsistent results on the association between the risk of DM and antidepressants.^15^ Moreover, as far as we know, there have been no studies on these relationships in older adults with Alzheimer's disease. Although our study demonstrated that antidepressants were not associated with DM in patients with Alzheimer's disease, further studies are necessary to determine the role of antidepressants on DM. Despite a few studies which reported that sedative anxiolytics might increase metabolic risk in the elderly, our results also showed no association between sedative anxiolytics and the risk of DM.^15^

Our study demonstrated the following strengths. This study integrated patients’ clinical data from a multicenter hospital with medical insurance claim data. We could thus use important clinical information related to development of DM, such as disease severity and BMI that could not be accessed by medical claim data. We also could minimize the possible miscoding of diagnoses by using CREDOS database.

The study also had some limitations. First, since we focused on any exposure to each psychotropic drug, the cumulative effect on the development of DM of each psychotropic drug was not examined, which were measured by cumulative duration and dose of each drug. Second, we could not proceed with subspecialized evaluation of each psychotropic drug because of the small numbers of subjects receiving each psychotropic drug. Third, the subjects who participated in this study were not systematically randomized according to psychotropic drugs. Fourth, this study did not control other DM-associated factors, including medications such as beta adrenergic antagonists, glucocorticoid, valproate, carbamazepine or thiazide, history of impaired glucose tolerance, smoking, family history of DM, history of gestational DM, and sedentary lifestyle reported by the American Diabetes Association.^38^ Fifth, we did not consider whether the use of psychotropic drugs were monotherapy or combination therapy; thereby, we involved 3 types of psychotropic drugs in statistical analysis as covariates.

In conclusion, this study suggested that antipsychotics used in patients with Alzheimer's disease might increase the development of DM, while antidepressants and sedative anxiolytics did not. Elderly patients with Alzheimer's disease should hence be monitored for the risk of DM while receiving antipsychotics in clinical practice. This result provided further impetus to future studies on psychotropic drugs associated DM in older adults with dementia.
